# Improved staffing policies and practices in healthcare based on a conceptual model

**DOI:** 10.3389/fpubh.2024.1431017

**Published:** 2024-09-30

**Authors:** Ning Wei, Zhongwei Wang, Xiyuan Li, Yibing Zhang, Jing Zhang, Ziying Huang, Xiao Wang

**Affiliations:** ^1^Shenzhen Maternity and Child Healthcare Hospital, Shenzhen, China; ^2^Sichuan Clinical Research Center for Cancer, Sichuan Cancer Hospital & Institute, Sichuan Cancer Center, Affiliated Cancer Hospital of University of Electronic Science and Technology of China, Chengdu, China

**Keywords:** staffing management, personnel shortage, skills mismatch, hospital, conceptual model

## Abstract

**Background:**

Staffing policies are critical in healthcare facilities. However, problems from a mismatch between staff numbers requirements and offers are frequently encountered. This research examines the joint effects of quantitative and qualitative staff mismatch in a healthcare organization in China to understand how staffing management policies affect staffing adequacy and how staffing adequacy leads to important human resource (HR) outcomes.

**Methods:**

In a previous study, we identified four dimensions of staffing management policies, namely decision-making, data management, productivity optimization, and title vs. competency. Based on this categorization, an 11-item scale was generated and evaluated for psychometric quality. A quantitative study was conducted with 1,323 healthcare professionals (including clinical and administrative staff) working at the hospital, matched by dyads and teams. A conceptual model with work engagement as a mediator between quantitative staffing adequacy interactions and qualitative staffing adequacy was tested with structural equations.

**Results:**

The findings indicate that staffing policies do exert effects on staffing adequacy. These findings further indicate that quantitative and qualitative staffing adequacy interact in explaining work engagement and team performance and that the moderated mediation occurs as hypothesized.

**Conclusion:**

Our findings indicate that both types of staffing adequacies contribute to higher team performance via a heightened sense of work engagement from healthcare professionals. Furthermore, a modulation is observed between the two types of staffing adequacies during the promotion of higher team performance. The supported model is helpful in improving staffing management policies and increasing staffing fit so as to improve hospital performance.

## Introduction

Since the founding of New China, especially after the implementation of the “Reform and Opening” policy, China’s healthcare has made world-renowned achievements, with a significant increase in the average life expectancy of people (1), a significant decrease in neonatal mortality, and a gradual improvement in various health indicators (2).

In recent years, China’s healthcare sector has continued to develop at an astonishing rate, with spending increasing from US$ 357 billion in 2011 to US$ 1 trillion in 2020 (3). From pharmaceuticals to medical products to consumer health, China remains one of the world’s most attractive markets globally. Three themes that shape China’s healthcare market are the continuation of economic and demographic trends, further healthcare reform, and the policies articulated in the government’s 12th five-year plan back in 2010 (4). Some of these forces, such as improvements in infrastructure, the broadening of insurance coverage, and significant support for innovation, have positive implications for multinational companies. Others, such as the pressure on pricing and the rise of local champions, may have negative implications. In some respects, including the bid to reconcile low-cost universal healthcare coverage with rewards for innovation, the forces come into direct opposition.

Similar to many other countries, China’s healthcare reform has also undergone a difficult exploratory process. China’s healthcare reform has been divided into three stages (5): Stage 1: 30 years after the founding of the People’s Republic of China (1949–1979); Stage 2: 30 years after the “reform and opening up” policy (1979–2009); and Stage 3: the latest round of healthcare reform (2009 to present).

In stage 1, with a weak foundation, the state developed a centrally planned socialist system, emphasizing public ownership and welfare, mass-based collectivism, and egalitarianism. Similar to the situation in Ethiopia, as discussed by Gile et al. (6, 7), public hospitals had little room for organizational-specific human resource management (HRM) measures owing to strict government regulations and control. In stage 2, China initiated a “reform and opening-up” policy, which ushering in a socialist market economy encouraged a free market and focused on economic growth. In stage 3, with the policy objective of achieving a “harmonious society” as a national priority, the Chinese government launched a fresh round of healthcare reform in 2009. It is an unprecedented health system transformation toward Universal Health Coverage. Following extensive interagency consultation and public debates, this launch emphasized a return to government-led, people-centered healthcare and healthcare as a public good. The latest round of healthcare reform adopted the “best fit” with the existing institutional and policy frameworks toward achieving Universal health coverage (UHC) by an incremental approach (step-by-step), which was recommended by the World Health Organization (WHO) team.

After a long-term development, the Chinese healthcare system has grown into a comprehensive and multi-level system. China has established the world’s largest medical security network, covering 1.36 billion people, with a stable participation rate of over 95%, thereby achieving universal coverage of the basic medical insurance system. In the last two decades, the Chinese government has further reformed the healthcare system. The establishment of the National Healthcare Security Administration in 2018, marking a new phase in the reform of the healthcare security system, has implemented measures such as centralized quantity-based procurement to reduce drug prices, adjusting the list of medical insurance drugs, and promoting the reform of medical insurance payment methods to ensure the welfare of the people (8). As reported by the Chinese government, the number of medical institutions and hospitals in China (including both public and private hospitals) has exceeded 1.03 million and 36,000, respectively. Furthermore, the number of medical personnel has also significantly increased, with over 11.24 million health technicians in 2021 (9). According to a series of policies such as the Outline of the “Healthy China 2030” Plan for the massive health industry, by 2030, China’s per capita life expectancy will be 79.0 years old, infant mortality rate will be 5.0%, and the total scale of the health service industry will reach 2.27 trillion USD (10).

Despite these internationally recognized achievements, China’s healthcare system, like any other, is subject to dynamics that are intrinsically linked to population changes (e.g., aging, birthrate) and pose new challenges and issues that have been progressively tackled with the deepening of the reform of the medical and health system (11). These challenges are currently surfacing in the area of healthcare human resource management, which gained central importance in 2009 when they were formally targeted as a priority in policymaking (12). One of the challenges in this domain pertains to the practices concerning how to achieve alignment between staffing needs and staffing practices in healthcare organizations.

China’s health human resource management is a comprehensive and systematic project. In terms of personnel allocation, it is required by the government that the number of practicing (assistant) physicians should reach 2.5 per thousand permanent residents, the number of registered nurses should reach 3.14 per thousand permanent residents, the medical-to-nursing ratio should reach 1:1.25, the bed-to-nursing ratio of municipal and above hospitals should not be less than 1:0.6, and the number of public health personnel should reach 0.83. China implements a personnel mechanism for public institutions with employment and job management systems as the main content. It also improves job setting management and ensures that professional and technical positions occupy a significant proportion. Enhance the reform of the income distribution system by establishing an assessment and incentive mechanism that is centered on service quality, service quantity, and satisfaction of service recipients (13).

Furthermore, the 14th Five-Year Plan for the Development of Health Talents released by the government sets goals such as steady growth in the total amount of health talent resources, further optimization of talent structure and regional distribution, and further improvement of talent service capabilities. The emphasis was placed on key tasks such as improving the level of high-end talent aggregation and innovating and improving talent management systems (14).

The introduction of management policies for healthcare human resources (HR) in China has promoted the construction of talent teams. However, the management of healthcare human resources in China still faces major challenges despite healthcare human resources in China having been greatly improved by management policies, as noted above. Staffing is always a complex issue in management because it relates to a dynamic reality that is not always within the predictive ability of decision-makers. These dynamics express changes in the staff that make, e.g., employee turnover a guaranteed phenomenon for many reasons, such as retirement, voluntary quitting, transfer, sickness leave, or any other motive that may escape the control of managers. As the content of the job becomes more complex, more staff will be needed, and the staff needs to have a greater range of competencies. Staffing decisions also become more complex and difficult, which requires greater insight into the staffing management field.

There are two aspects of staffing that decision-makers need to consider: quantity and quality. In terms of staffing quantity, both too much and not enough staffing are undesirable situations. Only an optimal outcome will lead to improved productivity, as evidenced by the World Health Organization issue of the Global Strategy on Human Resources for Health: Workforce 2030. This global policy is designed to counter the estimated shortage of 10 million health workers by 2030. This has been stated to be the biggest threat to global health because understaffing has been found to lead to critical outcomes related to patient risk, treatment effectiveness, and preventable death. On the other hand, the lack of qualified medical staff is a relatively common phenomenon worldwide. For example, approximately 6–12% of physicians in the United States do not meet the requirements of their jobs. Data from Ontario show that about 15% of family physicians and 3% of specialty physicians are not qualified for their jobs (15) and in the Netherlands, the estimated prevalence is 5% (16). Although researchers mention that staffing management has both a quantitative and a qualitative misfit situation, the coexistence of both types of staffing misfits has not been empirically studied.

## Literature review and hypothesis

The use of HRM, both “soft” and “hard,” to improve the performance of employees, teams, institutions, and patients in healthcare facilities has been extensively discussed in various kinds of literature (6, 17), while staffing is arguably the most important practice in HRM because it is a fundamental feature that, if badly achieved, will not be compensated by training, incentives or communication activities in the organizations (18). Therefore, we can also notice that staffing consists of two dimensions (19): the number of employees and the quality of employees, namely, quantitative and qualitative staffing.

Staffing management policies are vital because they not only improve hospital quantitative and qualitative staffing inadequacy but also put forward high demands for human resources. Several factors may explain why wrong staffing management policies may lead to quantitative staffing inadequacy, including staff burnout, employee resignation, and insufficient job satisfaction.

Freudenberger (20) employed the novel concept and term of “staff burnout” to explain individuals such as medical and health staff and social staff. The long work hours and high labor intensity are evident in the body and spiritual formation. The development process of burnout can be divided into three stages (anxious-depressive symptoms, resistance stage, and exhaustion) according to a theory developed by a Russian researcher (21). As Stoyanova and Harizanova (22) observe, this theory was developing simultaneously and independently from Maslach’s study (23, 24) with a striking convergence in signals used to detect it: emotional exhaustion, depersonalization, and loss of personal accomplishment.

Beeber (25) illustrates the impacts of nurse delegation policies on staffing and service availability. Psychological intervention is biased toward work careers, lacking a scientific theoretical foundation and many experimental research results. During this period, different researchers initially studied questions from corporate employees from various perspectives. Although there was no systemic coherence, it laid the foundation for in-depth research on the diversified research of corporate employees in the future. Maslach and Pines (23) provide three modes for corporate employee questionnaire surveys, namely emotional exhaustion, dehumanization, and lowering corporate feelings, which have a universal and profound social impact. However, implementing psychological intervention services encountered obstacles, as medical staff were reluctant to participate in group or individual psychology interventions (26).

It is therefore not surprising that the consequences of ill-defined staffing policies may trigger detrimental effects on employees. These policies may originate from a shortage of personnel, which translates into a higher workload for each individual. This excessive workload creates stress, negative events, and a negative working environment that can trigger the burnout process. A study conducted by Gutsan (27) found a direct effect of the nurse-to-patient ratio on the psychological health and productivity of nurses, which also affects the patient’s health.

It is well documented that burn-out employees are more prone to voluntarily abandon organizations (28) and also in search of a better work-life balance (29) or a more meaningful job (16). Policies, expressed as perceived organizational support, may also be conceived as exerting not a direct effect but rather an interaction effect in cushioning negative effects from work stress (30).

Therefore, we hypothesize, based on the influences of burnout and satisfaction, that.

Hypothesis 1: Staffing management policies impact the level of quantitative and qualitative staffing inadequacy.

Due to the healthcare staff shortage concomitant with the mounting numbers of patients and the difficulty of medical treatment, the workload is expected to increase, especially in contexts such as COVID-19 where emergencies are the rule and not the exception. The demand for nursing staff is higher, and the workload of nursing staff is also larger in such circumstances. Such a workload has potential consequences that are serious and unfavorable, along with long work shifts and low control. These have been found to lead to burnout, which harms staff and patients (31). Research has shown that high loads and long workloads are associated with nurses’ reduced productivity, complaints of fatigue, headaches, and susceptibility to illness, as well as feelings of depression, negativity, and sadness among nurses, which affect not only individual nurses but the entire team (32). Some scholars have also pointed out that in a high-stress environment, it is also necessary to reduce work demands and increase work resources to promote long-term work engagement and reduce emotional exhaustion (33, 34). The research found that inadequate staffing levels, workload, and working in a hurry may increase the risk of omissions and other types of error, as well as patient harm (35). Additionally, other scholars have pointed out that daily exposure to environments with high workload/staffing ratios is associated with an increased risk of death in critically ill patients and suggested that staffing should be based on workload, not just the number of patients, and even “fixing” fewer nurses for a short period, or a temporary increase in Intensive Care Unit (ICU) capacity without a commensurate increase in staffing, may adversely affect patients (36).

Furthermore, the excessive workload changes the internal psychological perception, which depletes motivation and leads to a decline in motivation and enthusiasm for work, that is, the decline in work engagement and enthusiasm, which negatively impacts their performance (37). This is confirmed in research, as researchers found that the eight key characteristics that best predict job burnout are overwork, understaffing, administrative burden, professional relationships, organizational culture, values and expectations, intrinsic motivation, and work-life integration (38). Although the negative effects of quantitative staffing insufficiency on healthcare professionals themselves are enough to warrant attention, the extended negative consequences make this topic even more critical. Such is the case of understaffing severely impacting patient safety, quality of care, and staff outcomes (39). High turnover rates, inadequate staffing levels, increasing workload and high stress levels have been found to hamper the ability to provide high-quality patient care (40). This is also related to the staff’s deteriorating motivation, which spills over into the lower quality of care and team performance levels (41).

From the perspective of resources, some studies regard the number of employees as the resources within the organization to explore the impact of employee layoffs on organizational performance (19). When the quantitative staffing level in the organization is insufficient, it can be considered that the resources in the organization are in a state of lack, and therefore, the work performance will be negatively affected and reduced.

Thus, the following hypothesis is formulated:

Hypothesis 2: Quantitative Staffing Inadequacy is negatively associated with perceived team performance.

All the reported relations between quantitative staff management (inadequacies) and negative outcomes, namely job performance (and subsequently, organizational performance), gather consensus among scholars that have researched this topic. However, the explanatory mechanism is still diverse, as some authors highlight workload pressure and eventually resource depletion [leading to burnout (27)] others highlight the individual perception of not being considerate by managers due to a lack of support (42) but a strong intervening variable that links staffing inadequacy and job performance (or any other level of performance, team or organizational) is needed for further research. We believe, from the literature review, that quantitative work engagement is a suitable construct to bridge staffing inadequacy to performance.

Work engagement refers to a positive and complete emotional and cognitive state related to work (43), and it is the basic job requirement for employees in many enterprises or professions. The influencing factors of work engagement include individual characteristics such as psychological security and self-efficacy (44), as well as job characteristics such as the richness of work content and the importance of work (45), as well as factors related to the organizational environment, such as the management regulations related to the organization and the organization’s attention to individuals.

Logically, quantitative staffing inadequacy may exert an important effect on the organizational environment that affects work engagement. Previous studies have shown that adequate staffing and a balanced workload are essential for achieving good outcomes (46), which emphasizes the benefits of appropriate personnel allocation. Conversely, quantitative staffing inadequacy may increase the workload of healthcare workers and lead to poor outcomes. Stress caused by a high workload can negatively affect work engagement. Specifically, the high intensity of work can take a toll on healthcare workers both physically and psychologically.

For example, Sathiya (47) believes that the pressure of doctors and nurses is common and a worldwide problem. They found that insufficient personnel and resources are one of the important stressors when assessing the prevalence and sources of perceived pressure from doctors and nurses. Overloaded work may cause too much pressure on medical staff and make them ignore some tasks, or they may not be able to complete tasks on time, which will cause great harm to work engagement. There is supporting evidence from the literature. Cai (48) discussed the role of workload and occupational stress in predicting work engagement. They believed that organizational factors, such as workload, would have an impact on employee engagement. They emphasize the importance of ensuring that staff are maintained at adequate levels both qualitatively and quantitatively in order to prevent depletion of personal energy and to protect staff from exhaustion. The authors argue that only in this manner can healthcare workers cope with the demands of the job. Using a two-level structural equation approach, Ancarani (49) investigates the links between organizational climate and work engagement in a sample of public hospitals in Italy. Drawing from the Job Demands-Resources model, there is a positive association between work engagement and a climate that promotes workers’ autonomy, empowerment, and well-being, whereas it suggests that a climate based on efficiency and goal attainment is not favorable for engagement.

van Zyl (50) proved through empirical research that excessive workload leads to emotional exhaustion. Emotional exhaustion can lead to job burnout among medical staff and then adversely affect their work engagement. Job burnout is a psychological concept that describes a long-term emotional exhaustion, physical fatigue, reduced work involvement, a cold attitude toward clients, or a low sense of achievement at work.

Revisiting Maslach’s definition of job burnout as a comprehensive state, including emotional exhaustion, deindividuation, neuroticism, and low personal achievement, caused by the inability of service industry practitioners to effectively cope with the continuous pressure at work, we can infer that anything that depletes psychological resources may also deplete a sense of being engaged at work (51).

Kim (52) proposed that job burnout could be explained as an antecedent variable affecting work engagement. It can be concluded that quantitative staffing inadequacies may increase the workload of healthcare workers and make them feel stressed and burnt out, which may negatively affect their level of work engagement.

By integrating the construct of engaging leadership in the job demands-resources model, Schaufeli (53) found, with a transversal sample that also has healthcare professionals, that job demands and job resources fully mediate the relationship between engaging leadership and work engagement and burnout.

In addition, the Job Demands-Resources is one of the most commonly used theories to explain work engagement (54, 55). Employees are more likely to engage in work when they are faced with high challenges and have sufficient work and personal resources to cope with them (56). Therefore, we can infer that when there is a quantitative staffing inadequacy, namely insufficient staff, healthcare workers lack sufficient resources to cope with the challenge of a heavy workload. They may therefore reduce their work engagement.

Furthermore, we can explain the relationship between quantitative staffing inadequacy and work engagement from the perspective of person-job fit theory (P-J fit). Person-job fit refers to the degree to which an employee’s characteristics match with their job characteristics. This includes the matching of their knowledge, skills, and abilities with the needs of the job and the matching of their needs with the characteristics of the job. Edwards (57) refined the measurement indicators of person-job fit, including the degree to which an enterprise requires employees to be consistent with their working hours, effort level, and related work skills. We can speculate that when quantitative staffing is inadequate, employees will take on more workload and require more working hours, which may not match their characteristics or willingness and then lead to low work engagement.

Based on the above analysis, we propose that:

Hypothesis 3: Quantitative Staffing Inadequacy is negatively associated with work engagement.

There are many mediating factors that contribute to the decline in nursing quality due to insufficient staffing of nursing staff, such as the ability to respond, the use of new technologies and working methods, the ability to monitor patients, and the occurrence of adverse events (58). However, most researchers focus on work in the concept of work engagement. This is in line with previous hypotheses that work engagement is suitably a mediating factor in the performance level decline caused by quantitative staffing inadequacy. Therefore, by joining both hypotheses 1 and 2 in the same model, we can reason that:

Hypothesis 4: Quantitative Staffing Inadequacy exerts a negative indirect effect on team performance via work engagement.

As stated, quantitative staffing inadequacies are the first type of staffing issue that arises in the minds of most people. It is without surprise that the majority of literature emphasizes this quantitative dimension of staffing. However, qualitative staffing inadequacies can occur even when hospitals have the correct number of people.

Qualitative staffing refers to the skills or qualifications necessary to exert the profession. Critical shortages of skilled staff constitute a significant bottleneck in providing timely and quality obstetric care, thereby significantly impacting maternal and neonatal outcomes (59). The lack of sufficient skilled staff plays a dual role in providing timely medical assistance to those in need (60). These authors proposed that there are three phases of medical service delay that could jeopardize the patient’s health. The delays are first due to the patient’s postponing seeking medical care, second due to the difficulty in accessing healthcare facilities, and third due to receiving such healthcare service in the aftermath of entering such facilities. This final phase of delay has been attributed to the few skilled staff available to carry out adequate care (61). This intersects with resource and equipment shortages that delay the onset of appropriate treatment and leave skilled staff unable to carry out their professional role or to operate to the required standard (62).

Therefore, a perfect staffing match to the needs requires the correct number (quantitative) of people and the right skills (qualitative). The intensity of nursing care, or the intensive effort spent at work, is important because staffing needs vary with the number of patients and the type of care provided for each patient. As nursing care intensity increases, the number of nursing staff required to care for patients properly will also increase (63). The factors that contribute to the level of intensity include (1) other human resources, such as support staff; (2) physical resources, such as unit layout; (3) the work design and technology, such as the level of computerization and nursing care model; (4) administrative practices; (5) the severity of the patients being cared for; and (6) the turnaround time to produce the product (patient turnover or throughput).

We, therefore, believe that the complete depiction of staffing adequacy in organizations requires both types of fit. Both quantitative and qualitative inadequacies can have an influence on job satisfaction and engagement. According to Kahn (64) and Kahn (65), a dynamic, dialectical relationship exists between the person who drives personal energies (physical, cognitive, emotional, and mental) into his or her work role, on the one hand, and the person who allows this person to express themselves. As stated previously, work engagement (66) is produced by both having sufficient psychological resources (among which skills as a tool to cope with tasks and emerging challenges) and reasonable work demands (such as workload, work intensity, and emotional stressors).

Departing from the idea that qualitative staffing adequacy will add up to quantitative staffing adequacy, we hypothesize that:

Hypothesis 5: The indirect effect of quantitative staffing inadequacy on team performance via work engagement interacts with qualitative staffing inadequacy in such a way that when qualitative staffing inadequacy is high the negative indirect effect is stronger, but when qualitative staffing inadequacy is low, the negative indirect effect is weaker.

This hypothesis entails two complementary interaction effects that, for parsimony’s sake, we chose not to formally state as hypotheses. These pertain to an interaction between qualitative staffing inadequacy in the relationship established in hypothesis 2 (qualitative staffing inadequacy interacts with the negative direct effect of quantitative staffing inadequacy on team performance in such a way that when qualitative staffing inadequacy is higher, the negative direct effect is stronger) as well as in the one established in hypothesis 3 (qualitative staffing inadequacy interacts with the negative direct effect of quantitative staffing inadequacy on work engagement in such a way that when qualitative staffing inadequacy is higher, the negative direct effect is stronger).

Overall, staff management policies play an important role in healthcare management because they can condition the staffing adequacy to the work demands, both as regards quantitative sufficiency and qualitative fit. If the quantitative staffing is insufficient, on the one hand, the external manifestation will result in a significant increase in the workload, which will make the staff feel too much pressure, cannot complete their work well, is not conducive to ensuring the quality of care, and even seriously threaten the safety of patients. Additionally, not having the right skills will increase the burden of having to perform tasks that require more effort and may also lead to more errors, which are detrimental to the performance of the individuals and therefore, their team. Better than trying to figure out what the solutions are to remediate the staffing inadequacies, such as rotating team job posts (67), it is wiser to prevent such a need to remediate by designing good staffing policies.

## Conceptual model

**Figure 1:**
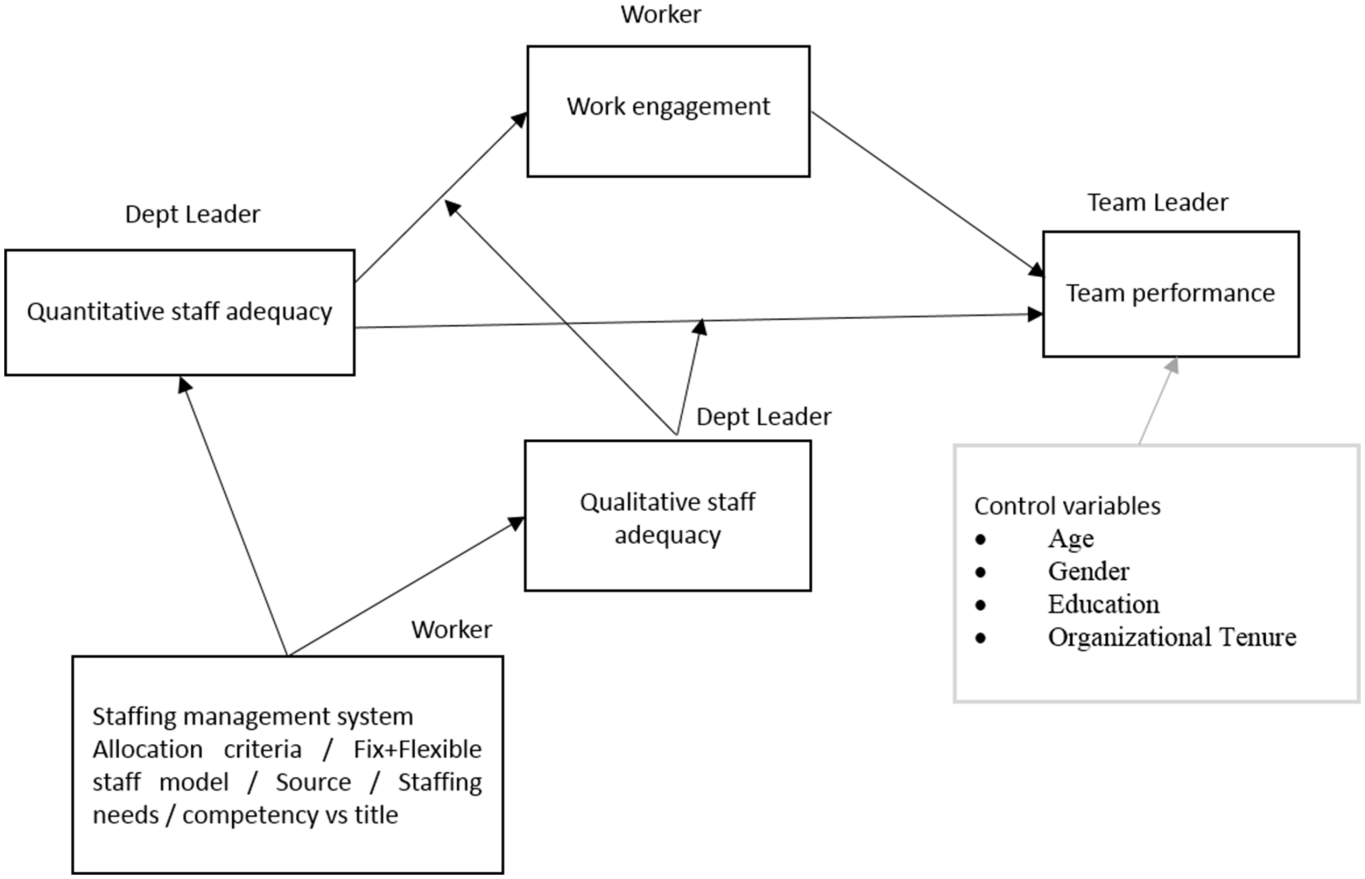
Conceptual model.

Based on the above theory and related literature, we propose a conceptual model involving five dimensions, including staffing management policy, quantitative staffing adequacy, qualitative staffing adequacy, work engagement, and team performance. Furthermore, based on important theories such as Burnout theory, Satisfaction factors, Atlantic Multidecadal Oscillation (AMO) Theory, Social Exchange Theory, Person-Post Matching Theory, and Job Demand-Resource Mode, we integrate the five hypotheses into a conceptual model shown in Figure 1.

The hypotheses are:

Hypothesis 1: Staffing management policies impact the level of quantitative and qualitative staffing inadequacy.

Hypothesis 2: Quantitative Staffing Inadequacy is negatively associated with perceived team performance.

Hypothesis 3: Quantitative Staffing Inadequacy is negatively associated with work engagement.

Hypothesis 4: Quantitative Staffing Inadequacy exerts a negative indirect effect on team performance via work engagement.

Hypothesis 5: The indirect effect of Quantitative Staffing Inadequacy on team performance via work engagement interacts with Qualitative Staffing Inadequacy in such a way that when Qualitative Staffing Inadequacy is high the negative indirect effect is stronger, but when Qualitative Staffing Inadequacy is low, the negative indirect effect is weaker.

## Methods

### Procedure

After approval to conduct a study on personnel allocation at Shenzhen Maternity and Child Healthcare Hospital was granted, the study received support from the hospital leadership, which has organized a mobilization meeting with the HR department to inform and facilitate data collection.

The survey was conducted at Shenzhen Maternity and Child Healthcare Hospital. The questionnaires in the printed version were distributed and collected on-site from staff in the post targeting first-level directors, first-level deputy directors, second-level directors, second-level vice directors, head nurses, team leaders, and regular team members (physicians, nurses, and other clinical and administrative staff). The research plan uses a census to conduct a questionnaire survey on 2,159 employees who have been employed at Shenzhen Maternal and Child Health Hospital for at least 1 year since we reasoned only those tenured more than 1 year would be able to answer as the perception of hospital management practices is not immediate and takes time to consolidate and become closer to reality. As our questionnaire is distributed on-site, some employees were not on duty (due to shifts, vacations, night shifts, etc.) when we visited the department for the survey. Therefore, we only surveyed employees who were on duty on the day of the questionnaire distribution and collected 1,600 questionnaires, accounting for 74.1% of all employees. This allows us to effectively eliminate random errors and ensure the reproducibility of the study.

### Data entry and data analysis strategy

The responses from each team member’s questionnaire were entered into rows and matched with the responses of the respective team leaders and first-level directors, resulting in a total of 1,260 items. Some departments had more than one team leader, but we only selected one of them to be included in the analysis. For example, the Galactophore department surveyed three team leaders, 2 first-level deputy directors (code: 028 and 029), and 1 head nurse (code: 030). We selected only 029 to be included in the analysis, so 65 team leader’s questionnaires were included. The criteria for selecting a leader, whenever there was more than one, was based on tenure. After data entry, the data were first checked for data entry quality and then cured to check for unusable answers due to monotonous answers or missing data, as well as for cases that did not have enough organizational tenure to be included in the analyses (less than 1 year).

With a workable database, we began by testing the psychometric quality of the measures, i.e., we tested the construct validity of all variables by confirmatory factor analysis (CFA), except for personnel management policies, for which we used exploratory factor analysis due to its tentative nature. CFA indicates the extent to which the data fit the theoretical structure initially proposed, and the fit indices used for judgments were based on the recommendations of Heinrich (68). To adopt cut-off points, we considered both the complexity of the model (based on the number of estimates) and the sample size, which in our case was significantly greater than 250.

According to best practice and recommendations, we adopted the normalized chi-square statistic (X^2^/df, also referred to as Chi-square Minimum Divided by Degrees of Freedom [CMIN/DF]), the comparative fit index (CFI), the Tucker-Lewis index (TLI), the root mean square error of approximation (RMSEA), and the standardized root mean residual (SRMR).

Fit indices are essential to judge the similarity between the estimated covariance matrix (the one theorized) and the observed covariance matrix (the empirical reality). The more mathematically similar the matrices are to each other, the better the fit of the theorized model. The null hypothesis states that there are no differences between those matrices.

One fundamental indicator of such similarity is the X^2^. This statistic is computed using Equation 2.1, where “N” stands for the sample size and the second term represents the differences between the observed model values and the proposed model values.

(2.1) $$x^{2}=f\left[\left(N-1\right)\left(S-\sum k\right)\right]$$

As the X^2^ formula implies that the value increases with increasing sample size, X2 is often biased upwards when using large samples. Therefore, with a larger sample, the *p*-value of X^2^ tends to decrease, thus rejecting the null hypothesis. For this reason, this statistic is often reported as Normed X^2^ (CMIN/DF), which is the ratio of the X^2^ to the degrees of freedom. Degrees of freedom are a measure of mathematical information and depend on the size of the covariance matrix, while they are immune to sample size effects. For this reason, the normed X^2^ index is a more robust index than the X^2^.

Another approach to fitting indices departs from the idea that instead of focusing on absolute values, it is preferable to compare the values obtained for the proposed model against a baseline model (e.g., the null model that assumes no correlation between latent variables or the saturated model that assumes a full correlated matrix). Such indices are called incremental fit indices, of which CFI is credited as an example.

Being a normed index, CFI values range between 0 and 1. Because it is less sensitive to sample size bias, it is often preferred over the X^2^ statistic. The formula is depicted in Equation 2.2, where “K” stands for the null model and “k” for the proposed model.

(2.2) $$\mathrm{CFI}=1-\frac{\left[X_{k}^{2}-{\mathrm{df}}_{k}\right]}{\left[X_{N}^{2}-{\mathrm{df}}_{N}\right]}$$

TLI is another incremental index, based on the comparison between CMIN for the null model (total absence of correlations) and the proposed model. Its formula is depicted in Equation 2.3.

(2.3) $$\mathrm{TLI}=\frac{\left[\left(\frac{X_{N}^{2}}{{\mathrm{df}}_{N}}\right)-\left(\frac{X_{k}^{2}}{{\mathrm{df}}_{k}}\right)\right]}{\left[\left(\frac{X_{N}^{2}}{{\mathrm{df}}_{N}}\right)-1\right]}$$

Statisticians have proposed a more sophisticated index to correct the sample size bias X^2^ is prone to: RMSEA. Its formula is depicted in Formula 2.4, where X^2^ for the statistical value of goodness of fit, df stands for the degree of freedom, “N” stands for the sample size and “k” stands for the proposed model.

(2.4) $$\mathrm{RMSEA}=\sqrt{\frac{\left(X^{2}-{\mathrm{df}}_{k}\right)}{\left(N-1\right)}}$$

Another complex fit index that deserves credit is the SRMR. This index is built on the reasoning that every covariance explained has a residual figure that expresses the error, and by standardizing such errors, one can identify cases that are deviating too much (conventionally taking |4| as a threshold), and by averaging these deviations and standardizing them, the index will express the magnitude of such residuals. Its formula is depicted in Equation 2.5, where the term within parenthesis represents the residuals comprehending the “S” as the sample matrix and the “I” as the proposed model matrix.

(2.5) $$\mathrm{SRMR}=\sqrt{\frac{1}{2}\sum {\left(s_{\mathrm{ij}}-I_{\mathrm{ij}}\right)}^{2}}$$

Therefore, we employed the following fit indices and their respective cut-off values. The CMIN/DF ratio should be below 3 and exhibit insignificant *p*-values; the CFI should be above 0.95, the TLI should be above 0.95, the RMSEA should be below 0.07, and the SRMR should be below 0.08.

As with all uses of indicators, the good practice is to use multiple so to benefit from their advantages and compensate for any disadvantages. Hair et al. (69) stress that the strictness with which one judges fit based on thresholds should take into account the sample size and model complexity. Stricter use should be applied to simpler models as well as to smaller samples. Likewise, alternative models should be compared so as to gauge the likelihood that the model has better grounds than a competitive explanation.

Our initial set of analyses pertaining to the hypotheses testing focused on the significance of staffing management policies played in explaining quantitative and qualitative staff adequacy. To evaluate this, we conducted a multiple regression analysis where the staffing management policies were given the status of predictor variables to explain quantitative staff adequacy in a first analysis and then qualitative staff adequacy in a final analysis (both taken as dependent variables).

To test the direct, indirect, and interaction effects previewed in the conceptual model, we ran Hayes Model 8, which depicts the exact relationships we want to test. This model evaluates a moderated mediation where the interaction effects are expected to occur between the predictor (quantitative staffing inadequacy) and the mediator variable (work engagement) as well as between the predictor and the dependent variable (perceived team performance). We included as correlates the sociodemographic variables, namely: age, gender, organizational tenure, and education.

### Sample

The sample comprises 1,323 healthcare professionals from Shenzhen Maternity and Child Healthcare Hospital with at least 1 year of experience in the organization. The respondents have different levels of responsibility, with 53 being first-level directors, 65 being team leaders, and the remaining 1,205 being clinical and administrative staff. This sample has a response rate of 89.75%, which is well within the range of good representation. This scenario occurs at all levels of hierarchical involvement, as 53 of the 65 directors were involved (81.5%), and 123 of the 130 s-level directors were involved (94.6%) in this study. However, this last category was cut down to 65 workable answers due to a dyadic mismatch with the corresponding employee-level answers (as some departments can be small).

The sample is mostly female (82.3%), young (72.2% below 40 years old, with an average falling in the 30–39 years old range), and educated (27.4% higher education levels), and working in the organization for an average of 9.1 years (standard deviation [SD] = 7.43). A detailed sample is provided in Table 1.

**Table 1:** Sample description.

		1st level (directors)	2nd level (team leaders)	3rd level (staff)
Sample size		53	65	1,205
Mean age (SD)		50.31 (6.81)	49.22 (6.65)	2.02 (0.888)
Age range	≤29	0%	0%	30.7%
	30–39	5.6%	9.23%	43.8%
	40–49	35.2%	41.54%	19.1%
	50–59	59.2%	49.23%	5.7%
	≥60	0%	0%	0.7%
Mean tenure		20 (9.3)	20.0 (8.7)	9.1 (7.4)
%Higher education		64.8%	38.5%	27.4%

### Measures

Staff adequacy was measured with Hudson and Shen (70) understaffing scale comprising two dimensions: quantitative understaffing (three items, e.g., “There are not enough employees in our work unit to complete all required job tasks”) and qualitative understaffing (three items, e.g., “Our work unit needs employees with different skills from those the group currently possesses”) together with one item to check for overstaffing, both quantitative overstaffing (Considering the required job tasks and work volume, my work unit has too many employees) and qualitative overstaffing, also called, overqualification (People in my work unit generally have an education level above the requirements of the professional title or tasks they are doing). First-level directors were invited to respond to a 5-point Likert scale, wherein 1 signifies Strongly Disagree and 5 signifies Strongly Agree.

A CFA of this two-factor solution showed poor fit indices and issues pertaining to covariance matrix errors. We have thus conducted a Principal Component Analysis that showed reasonable validity indices (Kaiser-Meyer-Olkin [KMO] = 0.570, Bartlett’s X^2^(15) = 2,187, *p* < 0.001) with two factors accounting for 63.1% of variance after rotation (Varimax), but the items from the qualitative staff adequacy had too few commonalities. After removing these items, we found a two-factor solution (KMO = 0.602, Bartlett’s X^2^(6) = 1,774, *p* < 0.001) accounting for 81.9% variance, where the second factor comprises a single item. The first component comprises the three original items measuring quantitative staff adequacy and has good reliability (Cronbach alpha = 0.838) as well as convergent validity (Average Variance Extracted [AVE] = 0.683). As the correlation between the first component and the singular item that represents qualitative staff adequacy is non-significant (*r* = 0.013, *p* = 659), there are no discriminant validity concerns with this solution (Table 2).

**Table 2:** Rotated matrix for Exploratory Factor Analysis (EFA) staff adequacy.

	1	2
QuanUS2	0.942	−0.030
QuanUS1	0.860	−0.019
QuanUS3	0.803	0.065
QualUS3	0.008	0.999
Cronbach alpha	0.838	–
AVE	0.683	–

Extraction Method: Principal Component Analysis.

Rotation Method: Varimax with Kaiser Normalization.

Rotation converged in three iterations.

Staffing Management Policies were measured using the scale originated from our previous study (a qualitative inductive study) and comprises 13 items distributed by five dimensions:

- Decision making (two items, “It is a good practice to allow departments of the hospital to decide on their own staffing needs,” and “It is a good practice to allow departments of the hospital to freely allocate work without a centralized decision making”),
- Data management (“It is better to collect data about staffing adequacy on the level of base workers rather than from the departmental director,” and “Any organization must have an integrated and efficient Information System to properly assess and monitor staffing levels”),
- Sourcing (one item, “It is a good practice to hire third-party temporary/rotating staff to complement work peaks,” plus a contingent item to the first one, if positive (sourcing is 4 or 5): “What percentage of the total staff should be third-party? ___ %”),
- Productivity optimization: whether the current productivity is optimal (4 items, “Considering the current manpower in the hospital I think we are using 100% of workers full productivity potential”, “The level of absenteeism in the hospital is not sufficiently high to harm its overall efficiency”, “The current level of workers motivation is good enough to promote high productivity”, and “The overtime practiced in the hospital falls within the reasonable number of hours and is not excessive”)
- Title vs. Competency (three items, “Competency, more than formal title, is used in the Hospital as the key criterion to decide on career promotions,” “Competency, more than formal title, is used in the Hospital as the key criterion to recruit and select new employees,” “Competency, more than formal title, is used in the Hospital as the key criterion for performance appraisal.”)

Team members were asked to respond to this scale using a 5-point Likert scale (1 = Strongly disagree to 5 = Strongly agree). For psychometric purposes, we have chosen to collect the answers from these participants as they comprise the largest sample, and this is a requirement to achieve a comfortable sample size to item ratio levels. This has the advantage of avoiding common source bias (71), because the staffing adequacy was collected from first-level directors.

A Principal Components Analysis demonstrated a solution that included one item with unacceptable commonality (Sourcing 1). We reason that this item has a distinguished nature from the remaining items and does not fit into a latent variable resulting from the shared variance of the remaining items. Removing this item showed a solution that could accommodate three to four components. We opted for the four-component solution due to theoretical reasons. The analysis revealed a valid solution (KMO = 0.802; 0.672 < MSAs < 0.874, Bartlett X^2^(55) = 4,805, *p* < 0.001) for 11 items, all with commonalities above 0.500. This explains 71.1% variance after rotation (Varimax), as shown in Table 3. All of the loadings were above 0.660, and the components were as follows: Productivity (four items, Cronbach alpha = 0.721; AVE = 0.791), Competency (Cronbach alpha = 0.905; AVE = 0.509), Data Management (Standardized Beta [rSB] = 0.622, AVE = 0.680), and Decision Making (rSB = 0.736, AVE = 0.725). Because the scale is novel, state reliability can be accepted for as low as 0.60.

**Table 3:** Rotated factor solution for staffing management policies.

	Component			
	1	2	3	4
Comp25	0.903	0.168	0.090	0.109
Comp26	0.891	0.152	0.112	0.118
Comp24	0.874	0.221	0.069	0.052
Produc22	0.166	0.794	0.091	0.014
Produc23	0.201	0.728	0.014	−0.093
Produc21	0.105	0.664	0.151	0.202
Produc20	0.094	0.660	0.230	0.115
DM16	0.113	0.156	0.852	0.148
DM15	0.097	0.191	0.851	0.123
DataM17	0.081	−0.048	0.179	0.842
DataM18	0.130	0.200	0.081	0.808
Cronbach alpha	0.721	0.905	0.736	0.622
AVE	0.791	0.509	0.725	0.680

Extraction Method: Principal Component Analysis.

Rotation Method: Varimax with Kaiser Normalization.

Rotation converged in five iterations.

**Figure 2:**
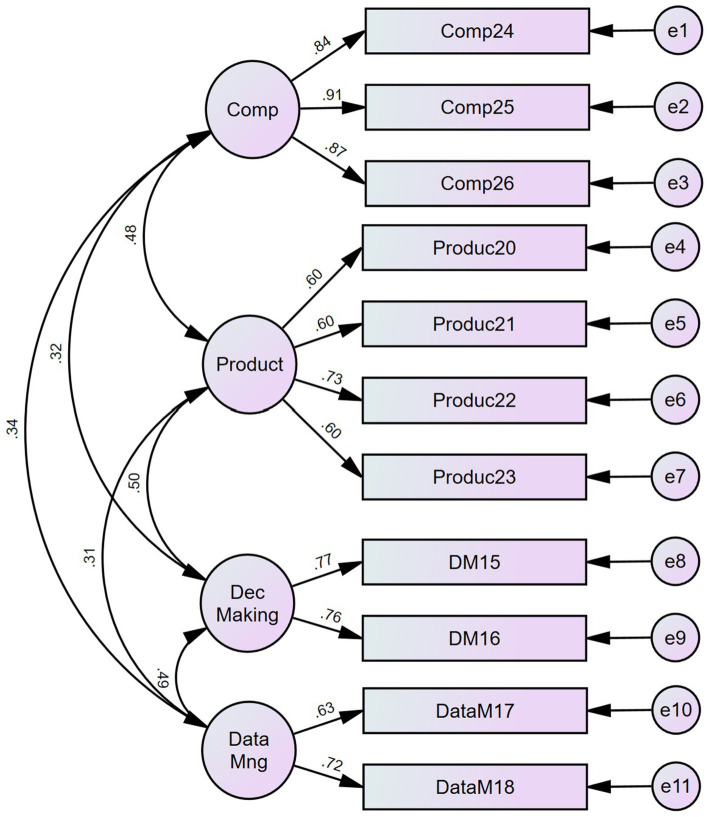
CFA for staffing management policies.

A CFA conducted on this factor solution showed good fit indices (Normed X^2^(37) = 4.52; CFI = 0.971; TLI = 0.959; RMSEA = 0.054 CI90 [0.046; 0.062] PCLose = 0.194; SRMR = 0.0381; Holter (*p* = 0.05) = 372). Figure 2 shows the CFA factor loading and structure.

Subjective team performance was measured with a scale [Conger et al. (72)] comprehending five items (“1. The workgroup I supervise has high work performance.”; “2. In the workgroup, I supervise most of our tasks are accomplished quickly and efficiently.”; “3. Workers in the workgroup I supervise always set a high standard of task accomplishment.”; “4. The workgroup I supervise always achieves a high standard of task accomplishment.”; and “5. The workgroup I supervise almost always beats our targets.”)

**Figure 3:**
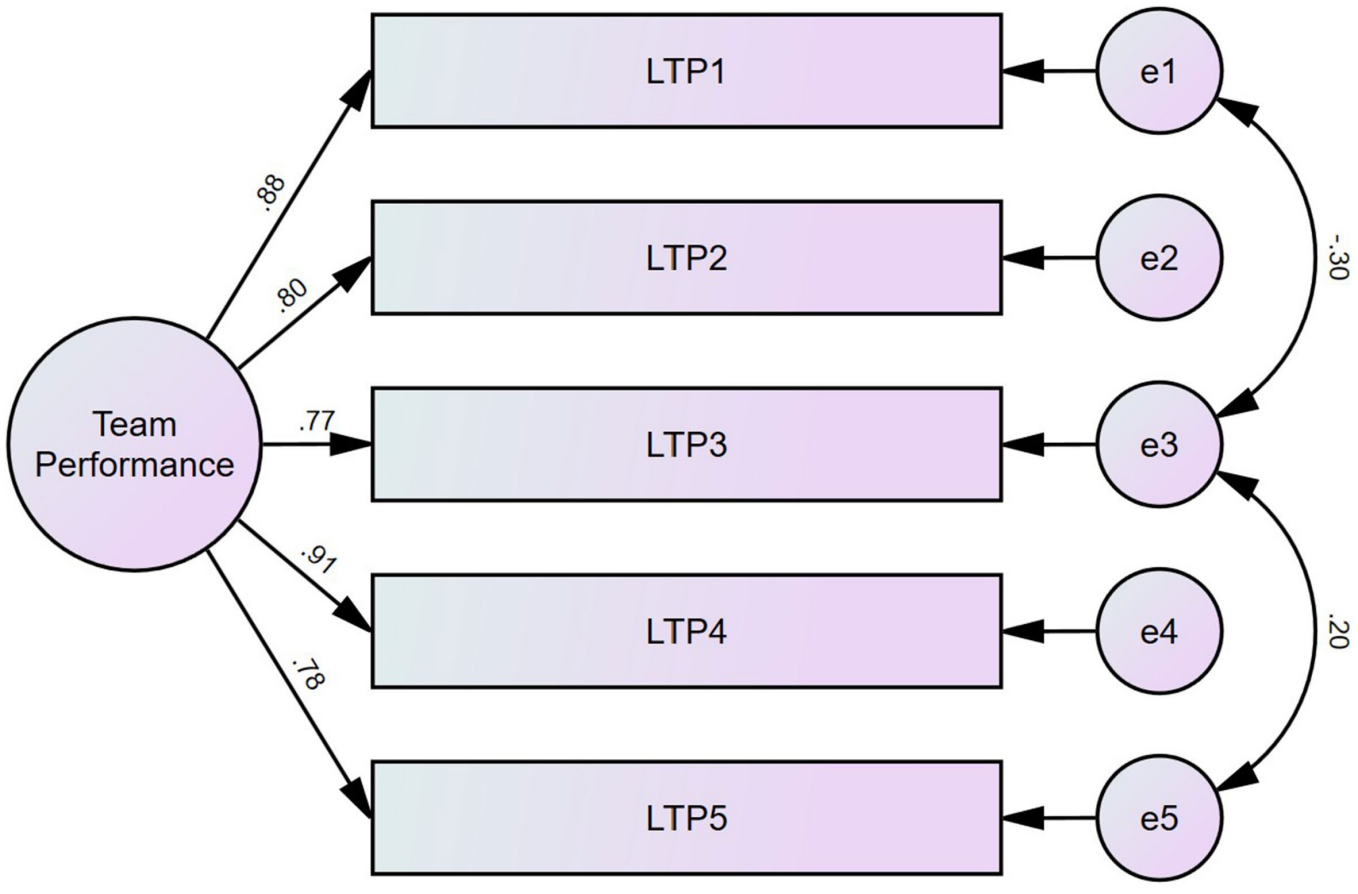
CFA for team performance as stated by team leaders.

Team leaders were asked to respond to this scale using a 5-point Likert scale (1 = Strongly disagree to 5 = Strongly agree). The Confirmatory Factor Analysis of the single factor solution revealed issues with RMSEA (0.109, CI90 [0.089; 0.132] PCLose = 0.000), and Lagrange multipliers suggested a covariance between the errors of the LTP3 and both Team leader subjective team performance (LTP) 1 and LTP5 (they are indeed very similar), and the resulting factor solution has good fit indices (Normed X^2^(3) = 7.55; CFI = 0.995; TLI = 0.985; RMSEA = 0.074 CI90 [0.047; 0.103] PCLose = 0.068; SRMR = 0.0113; and Holter (*p* = 0.05) = 416). This solution has excellent reliability (Correlation Ratio [CR] = 0.917) as well as high convergent validity (AVE = 0.689). Figure 3 shows the CFA factor loadings and structure for this sample.

**Figure 4:**
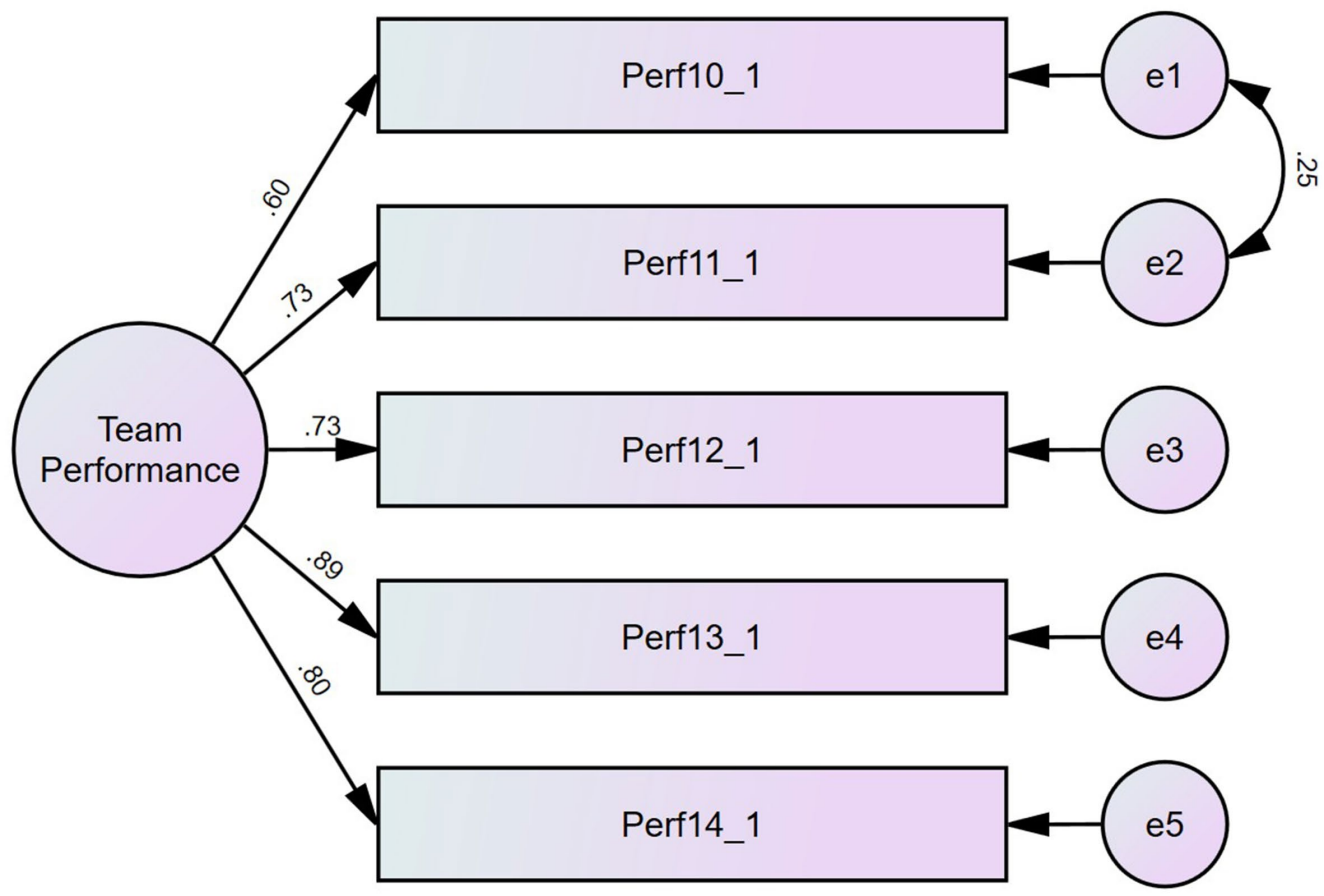
CFA for team performance as stated by team members.

Additionally, team members were asked to respond to this scale using the same answering 5-point Likert scale. The Confirmatory Factor Analysis of the single factor solution revealed issues pertaining to RMSEA (0.109, CI90 [0.089; 0.132] PCLose = 0.000) and Lagrange multipliers suggested a covariance between the errors of the first couple items (they are indeed very similar). The resulting factor solution has good fit indices (Normed X^2^(4) = 4.262; CFI = 0.996; TLI = 0.989; RMSEA = 0.052 CI90 [0.028; 0.079] PCLose = 0.398; SRMR = 0.0124; and Holter (*p* = 0.05) = 671). This solution has both good reliability (CR = 0.867) and high convergent validity (AVE = 0.571). Figure 4 shows the CFA factor loadings and structure for this sample.

Work engagement was measured using the Utrecht Work Engagement scale, which contains nine items distributed equally by three dimensions: vigor (“At my job, I feel strong and vigorous,” “At my work, I feel that I am bursting with energy,” “When I get up in the morning, I feel like going to work”), dedication (“I am enthusiastic about my job,” “My job inspires me,” “I am proud of the work that I do”), and absorption (“I feel happy when I am working intensely,” “I am immersed in my work,” “I get carried away when I’m working”).

**Figure 5:**
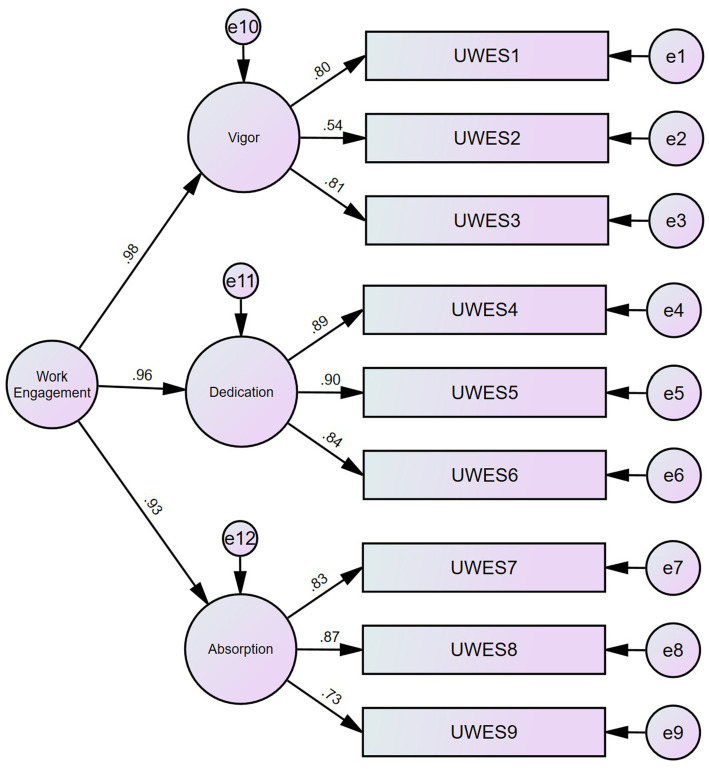
CFA for work engagement.

The team members were invited to respond on a 7-point frequency scale (0 = Never, 1 = Almost never/A few times a year or less, 2 = Rarely/Once a month or less, 3 = Sometimes/A few times a month, 4 = Often/Once a week, 5 = Very often/A few times a week, 6 = Every day). The Confirmatory Factor Analysis of the first-order factors suggested a second-order factor due to high interaction covariances. The first-order factors are psychometrically sound, as indicated by convergent validity (AVEvigor = 0.503; AVEdedication = 0.767; AVEabsorption = 0.661) and reliability (CRvigor = 0.746; CRdedication = 0.908; CRabsorption = 0.853). The second order factor solution showed good fit indices (Normed X^2^(22) = 6.766; CFI = 0.984; TLI = 0.973; RMSEA = 0.069 CI90 [0.059; 0.080] PCLose = 0.001; SRMR = 0.0207; Holter (*p* = 0.05) = 275). The second-order factor exhibits high reliability (CR = 0.97) and convergent validity (AVE = 0.92), as shown in Figure 5.

The control variables included the general sociodemographics, namely: Age range (1 = <29, 2 = 30–39, 3 = 40–49, 4 = 50–59, 5 = 60+), Gender (1 = Female, 2 = Male), Organizational tenure (for how many years have you been working here?) Education (1 = Less than 12 years of schooling, 2 = Secondary (12 complete years of schooling), 3 = Post-secondary (Zhuanke), 4 = Bachelor, 5 = Master, 6 = Doctorate/PhD).

## Results

This section begins by showing the descriptive and bivariate analyses to understand the extent to which the variables are present in the organization and what patterns of correlation they have. It will then show the findings of hypotheses testing showing goodness of fit for the structural equation models as well as the respective association coefficients (Lambdas) between staffing management policies and both quantitative and qualitative staffing adequacies. It concludes by presenting findings for the second part of the conceptual model regarding direct, indirect, and interaction effects, and respective interaction graphs whenever applicable.

### Descriptive and bivariate analysis

As shown in Table 4, descriptive statistics indicate that staffing management policies are mostly perceived as favoring competency instead of the title (mean = 3.66, SD = 0.81), decentralized decision instead of centralized (mean = 3.68, SD = 0.72), and root data collection at the employee level instead of departmental directors (mean = 3.98, SD = 0.60), and there is an opening to outsource staff to accommodate needs. Likewise, staffing issues (staffing adequacy, decision-making criteria, and data collection preferences) are perceived as being only moderately harming productivity (mean = 3.54, SD = 0.64). Work engagement is moderately high (mean = 4.51, SD = 0.80), and team performance is also similar (mean = 3.74, SD = 0.66 in a maximum possible of 5 points). As regards staff adequacy, no one in the sample strongly agreed that qualitative staff was inadequate, and the average is very close to the scale’s midpoint (2.5). Conversely, quantitative staffing seems to be acknowledged as an issue (mean = 3.56, SD = 0.80) as not a single participant strongly disagreed with the items expressing quantitative staff inadequacy, and about one quarter (23%) of the sample signaled 4 or more, thus agreeing there is a quantitative staffing issue.

**Table 4:** Descriptive and bivariate statistics.

	*N*	Min–Max	Mean	SD	1	2	3	4	5	6	7	8	9	10	11	12
1. Age	1,168	1–5	2.02	0.88	–											
2. Organisational tenure	1,075	1–39	9.06	7.43	0.736**	–										
3. Gender	1,205	0–1	0.18	0.48	−0.060*	−0.098**	–									
4. Education	1,205	1–7	4.78	1.26	−0.150**	−0.169**	0.086**	–								
5. SMP_Title vs. competency	1,200	1–5	3.66	0.81	−0.016	−0.021	0.053	−0.030	–							
6. SMP_Productivity	1,188	1–5	3.54	0.64	−0.050	−0.079**	0.071*	−0.042	0.402**	–						
7. SMP_Cent. vs. Decentr. decision	1,197	1–5	3.68	0.72	0.033	0.016	0.035	−0.009	0.262**	0.374**	–					
8. SMP_High level vs. root data	1,200	1–5	3.98	0.60	0.023	0.044	0.011	0.063*	0.243**	0.197**	0.335**	–				
9. Insourcing vs. outsourcing	1,205	1–5	3.83	0.80	−0.004	0.028	0.043	0.014	0.204**	0.205**	0.231**	0.361**	–			
10. Work engagement	1,185	1–6	4.51	0.80	0.021	−0.066*	0.043	−0.021	0.285**	0.480**	0.223**	0.125**	0.146**	–		
11. Quantitative staff inadequacy	1,205	2–5	3.56	0.80	0.023	0.029	0.006	0.063*	0.033	−0.082**	0.035	0.033	0.063*	−0.066*	–	
12. Qualitative staff inadequacy	1,205	1–4	2.54	0.93	−0.056	0.014	−0.037	−0.018	0.060*	−0.059*	0.012	0.042	−0.014	−0.081**	0.013	–
13. Team performance (team members)	1,190	1–5	3.74	0.66	−0.016	−0.034	0.074*	−0.066*	0.293**	0.574**	0.400**	0.218**	0.198**	0.553**	−0.077**	−0.065*

*p < 0.05; **p < 0.01.

As regards correlations, sociodemographic variables are not significantly correlated with the variables in the conceptual model. Age shows not a single significant correlation (*p* < 0.05), organizational tenure has only a minor correlation with work engagement (*r* = −0.06, *p* < 0.05), and gender has a suggestive correlation with the dependent variable, where masculine participants report slightly higher levels (*r* = 0.074, *p* < 0.05). More educated participants also report lower levels of perceived team performance, higher levels of quantitative staff inadequacy, and more agreement that staffing should consider data collection at the root level.

Staffing management policies have only light correlations with reported staff inadequacy, both quantitative and qualitative. The most relevant correlation occurs between productivity optimization and both dimensions of staff inadequacy and has a negative valence, meaning that the higher the optimization reported, the lower the staff inadequacy. Interestingly, quantitative and qualitative staff inadequacy have no correlation (*r* = 0.013, *p* > 0.05), suggesting that they have distinct realities within the organization. The strongest cases of correlations, all positive, occur with work engagement (ranging from *r* = 0.125, *p* < 0.01, and *r* = 0.480, *p* < 0.01) and team performance (ranging from 0.218, *p* < 0.01, and *r* = 0.574, *p* < 0.01). This suggests that staffing management policies may exert a positive effect upon work engagement and team performance. Mirroring this relationship, work engagement was found to negatively correlate with staff quantitative and qualitative staff inadequacy (albeit of a modest magnitude, *r* = −0.066, *p* < 0.05, and *r* = −0.081, *p* < 0.01, respectively). A strong correlation occurs between work engagement and team performance, thus encouraging the conceptual model. Overall, the pattern of associations suggests that the expected pathways in the conceptual model may have a sound basis.

### Hypotheses testing

The first part of the model examines the hypothesized relationship between staffing management policies and staffing inadequacies, both quantitatively and qualitatively. To evaluate the association between staffing management policies and quantitative and qualitative staffing, we have designed a structural equations model where the four staffing management policies (title vs. competency, productivity, decision-making, and data management) are treated as predictors of quantitative and qualitative staffing adequacy. Because these final constructs did not operate as latent variables, we opted to treat them as formative constructs, and therefore, we included as dependent variables in the model their composite index.

The Structural Equation Modeling (SEM) revealed a valid model with good fit indices (Normed X^2^ (83) = 3.21; CFI = 0.968; TLI = 0.941; RMSEA = 0.043 CI90 [0.037; 0.049] PCLose = 0.978; SRMR = 0.0381; Holter (*p* = 0.05) = 475) with significant estimates (*p* < 0.05) as shown in Table 5. After controlling for the effects of gender, age, education, and organizational tenure, title_vs_competency was positively associated with both quantitative staffing adequacy (*λ* = 0.102, *p* < 0.01) and qualitative staffing adequacy (*λ* = 0.111, *p* < 0.01). The same results were found for productivity (*λ* = −0.182, *p* < 0.001) for quantitative and (*λ* = −0.143, *p* < 0.01) for qualitative. Decision-making is significantly associated with only quantitative staffing adequacy (*λ* = 0.095, *p* < 0.05), and data management is not associated with any of these.

**Table 5:** Estimates for SMP-staffing adequacy model.

	Quantitative staff inad.					Qualitative staff inad.				
	Lambda	Estimate	S.E.	C.R.	*p*-value	Lambda	Estimate	S.E.	C.R.	*p*-value
Title vs. competency	0.102**	0.110	0.040	2.731	0.006	0.111**	0.139	0.047	2.973	0.003
Productivity	−0.182***	−0.268	0.070	−3.838	<0.001	−0.143**	−0.245	0.080	−3.049	0.002
Decision making	0.095*	0.123	0.063	1.973	0.049	0.029	.064n.s.	0.072	0.884	0.377
Data management	0.016 n.s.	0.027	0.079	0.336	0.737	0.016	0.056 n.s.	0.092	0.614	0.539
Age	0.007 n.s.	0.007	0.041	0.163	0.871	−0.126	−0.133**	0.047	−2.805	0.005
Gender	−0.063*	−0.132	0.064	−2.079	0.038	−0.053	−0.130 n.s.	0.074	−1.756	0.079
Education	0.060*	0.039	0.019	2.041	0.041	−0.028	−0.021 n.s.	0.022	−0.958	0.338
Organizational tenure	0.014 n.s.	0.001	0.005	0.299	0.765	0.073	0.009 n.s.	0.006	1.573	0.116

*p < 0.05; **p < 0.01; ***p < 0.001. S.E., Stands for Standard Error; C.R. Stands for Correlation Ratio.

These results indicate that the more individuals report that the hospital emphasizes competencies instead of titles, the more they tend to perceive insufficient numbers of staff and the need to hire different skill profiles of staff. Results also indicate that the more individuals perceive the hospital has optimized its productivity, the less they think that the hospital is lacking staff and lacking the right skills profile. Finally, the results also indicate that the more individuals perceive the hospital is decentralizing staffing decisions to the departmental level, the more they think there is quantitative staff inadequacy.

Overall, the findings support hypothesis 1, as staffing management policies are seemingly impactful in quantitative and qualitative staff adequacy, as reported by participants.

The second part of the model pertained to the process relationship that connects both quantitative and qualitative staff adequacies (treated as an interaction effect) to team performance via work engagement. For parsimony’s sake, we report all findings in Table 6. The table shows that, after controlling for gender, age, education, and organizational tenure, quantitative staff inadequacy harms perceived performance (*B* = −0.05, CI95 [−0.090; −0.004]), supporting hypothesis 2.

**Table 6:** Direct, indirect, and interaction effects.

Dependent variable	Work engagement						Perceived performance					
	B	S.E.	t	Boot LLCI	Boot ULCI		B	S.E.	t	Boot LLCI	Boot ULCI	
Direct effects												
Constant	4.24	0.20	21.09	3.84	4.63		1.57***	0.17	9.31	1.240	1.903	
Age	0.16***	0.04	3.84	0.080	0.248		−0.04	0.03	−1.35	−0.099	0.018	
Org. tenure	−0.02***	0.01	−4.24	−0.031	−0.011		0.00	0.00	0.83	−0.004	0.010	
Gender	0.09	0.06	1.44	−0.034	0.224		0.15**	0.05	3.25	0.059	0.238	
Education	−0.01	0.02	−0.62	−0.061	0.031		−0.02	0.02	−1.11	−0.051	0.014	
Quantit. staff inadeq.	−0.07*	0.03	−1.97	−0.135	−0.001	H3	−0.05*	0.02	−2.14	−0.090	−0.004	H2
Qualit. staff inadequacy	−0.04	0.03	−1.15	−0.094	0.012		0.00	0.02	0.03	−0.036	0.037	
Work_Engagement							0.44**	0.02	19.49	0.393	0.481	
Interaction effect												
QuantStaff*QualitStaff	−0.14***	0.04	−3.71	−0.215	−0.066		−0.09***	0.03	−3.55	−0.146	−0.042	H5
Single slope												
QualitStaff low												
QualitStaff high	0.06	0.04	1.69	−0.010	0.138		0.04	0.03	1.40	−0.015	−0.088	
Indirect effect	−0.20***	0.06	−3.39	−0.316	−0.084		−0.14***	0.04	−3.39	−0.220	−0.059	
QualitStaff low							0.03	0.02		−0.005	0.062	
H4 QualitStaff avr.							−0.03	0.02		−0.060	0.001	
QualitStaff high							−0.09	0.03		−0.139	−0.037	
Index moderated mediation							−0.06	0.02		−0.095	−0.037	
R2	4.3%						31.9%					

*p < 0.05, **p < 0.01, ***p < 0.001. S.E., Stands for Standard Error.

Similarly, the findings indicate that quantitative staff inadequacy is a predictor of work engagement (*B* = −0.07, CI95 [−0.135; −0.001]) with a negative valence, thus supporting hypothesis 3.

We also found that the relationship between work engagement and perceived performance was significant and positive (*B* = 0.44, CI95 [0.393; 0.481]), which suggests that the hypothesized indirect effect of quantitative staff inadequacy on team performance via work engagement is possible. However, the test showed a non-significant coefficient (−0.03, SE = 0.02, CI95 [−0.060; 0.001]), thus rejecting hypothesis 4. It is important to remember that the conceptual model theoretically previews a possible interaction effect with qualitative staffing inadequacy, and thus this finding is valid only for the unconditional statement.

**Figure 6:**
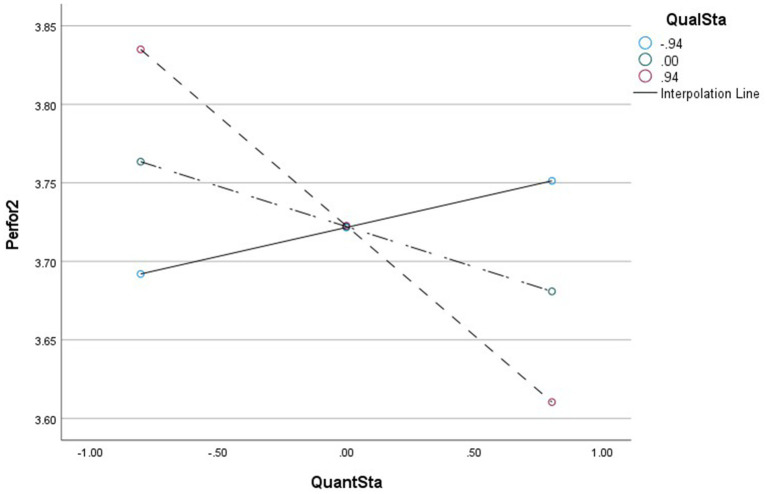
Moderating effect diagram QuanS*QualS->TeamPerformance.

As regards the interaction with qualitative staffing inadequacy, findings show quantitative staff inadequacy interacts with qualitative staff inadequacy, resulting in a significant negative effect on perceived performance (*B* = −0.09, CI95 [−0.146; −0.042]). Specifically, the relationship between quantitative staff inadequacy and perceived performance is significantly negative (*B* = −0.14, CI95 [−0.220; −0.059]) when the qualitative staff inadequacy level is high, while at a low qualitative staff inadequacy level, the relationship between quantitative staff inadequacy and perceived performance is not significant. This indicates that a high level of qualitative staff inadequacy will strengthen the negative effect of quantitative staff inadequacy on perceived performance. Figure 6 shows the interaction.

Furthermore, the results in Table 3 also show that qualitative staffing inadequacy interacts with quantitative staffing inadequacy in predicting work engagement (*B* = −0.14, CI95 [−0.215; −0.066]), which encourages the moderated mediated effect. When the qualitative staff inadequacy level is high, the indirect effect of quantitative staff inadequacy on perceived performance via work engagement is significant (*B* = −0.09, CI95 [−0.139; −0.037]), while the indirect effect is not significant (*B* = 0.03, CI95 [−0.005; 0.062]) when the qualitative staff inadequacy level is at average or at low levels.

**Figure 7:**
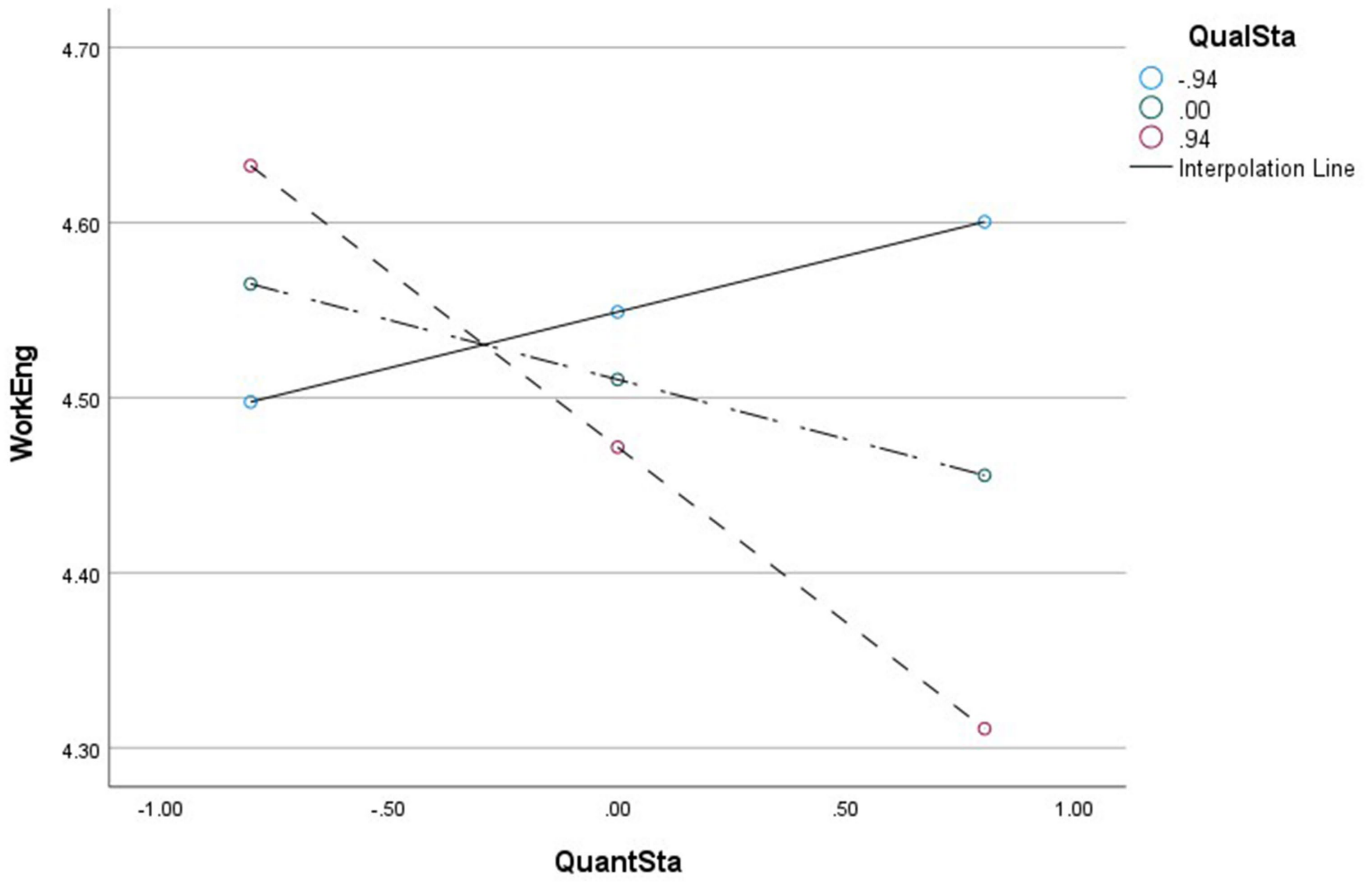
Moderating effect diagram QuanS*QualS->WorkEngagement.

This suggests that qualitative staff inadequacy moderates the relationship between quantitative staff inadequacy and perceived performance through work engagement, and that this negative relationship becomes stronger in the presence of a high level of qualitative staff inadequacy. Indeed, we further conducted a simple slope analysis to examine the disparity in the relationship between quantitative staff inadequacy and work engagement at various levels of qualitative staff inadequacy. When the qualitative staff inadequacy is at a high level, the relationship between quantitative staff inadequacy and work engagement is significantly negative (*B* = −0.20, CI95 [−0.316; −0.084]), while the relationship between quantitative understaffing and work engagement is not significant at a low level of qualitative staff inadequacy. This suggests that qualitative staff inadequacy modifies the relationship between qualitative staff inadequacy and work engagement. A high level of qualitative staff inadequacy and quantitative staff inadequacy has a stronger negative effect on work engagement. The interaction is depicted in Figure 7. Thus, the findings support the moderated mediation effect as stated in Hypothesis 5.

In light of these findings, we must consider that hypothesis 4 does not receive empirical support for its unconditional statement but that the indirect effect is conditional on the level of qualitative staffing inadequacy.

## Discussion and conclusion

Currently, the reform of China’s health human resource management policies permits medical institutions to operate with more autonomy, such as the de-staffing reform and the “two allowances” (allowing medical and health institutions to break through the current salary control level of public institutions and allowing medical service income to deduct costs and extract various funds according to regulations, mainly for personnel rewards) (73). The practice has proven that empowering medical institutions with more autonomy in human resource management is beneficial in balancing the mismatch between the quantity and quality of human resources. On the one hand, medical institutions can adjust the scale of recruitment according to their business development. On the other hand, establishing a robust but flexible management system (especially in terms of promotion/relegation and hiring and firing) is conducive to solving the problem of mismatched personnel and positions. These autonomy measures have enabled Chinese medical institutions to avoid the human resource crisis experienced in Ethiopia. Due to the government’s strict control over the size, number of employees, and salary structure of hospitals, it is difficult for hospitals to take targeted measures for their institutions, resulting in generally low satisfaction and motivation among doctors, as well as serious moonlight and resignations (7).

The Chinese government has adopted a strategy of national assessment to encourage medical institutions to carry out medical service projects and improve medical quality. Under this strategy, human resource allocation indicators are often instructive rather than mandatory, such as the ratio of personnel to beds, the ratio of doctors to nurses, etc. This approach enables primary medical institutions to better exert their subjective initiative and achieve their strategic goals. This strategy is undoubtedly more suitable for the national conditions of some countries compared to the strategies adopted by them (74), which makes medical institutions focus on the development of their comprehensive strength and avoids vicious competition among medical institutions (6).

This empirical study was designed to determine to what extent staffing management policies affect staffing adequacy (both quantitative and qualitative) and how these interact to produce a positive psychological status (work engagement), which leads, both directly and indirectly, to heightened work team performance in a Chinese healthcare context.

The conceptual model was then designed to test five hypotheses namely: H1 (Staffing management policies impact the level of quantitative and qualitative staffing inadequacy), H2 (Quantitative Staffing Inadequacy is negatively associated with perceived team performance), H3 (Quantitative Staffing Inadequacy is negatively associated with work engagement), H4 (Quantitative Staffing Inadequacy exerts a negative indirect effect on team performance via work engagement), and H5 (The indirect effect of Quantitative Staffing Inadequacy on team performance via work engagement interacts with Qualitative Staffing Inadequacy in such a way that when Qualitative Staffing Inadequacy is high, the negative indirect effect is stronger, but when Qualitative Staffing Inadequacy is low, the negative indirect effect is weaker).

Globally, the first hypothesis refers to the policy-to-practice process; the second, third, and fourth hypotheses refer to how quantitative staffing adequacy leads directly and indirectly to team performance; and the last hypothesis refers to how qualitative staffing adequacy should also be equated to fully understand how the process works.

Findings show the impact of staff management policies on human resource allocation is mainly reflected in the following three aspects: staff competency and title, productivity, and decision-making.

Regarding competency and title, leaders who tend to believe that actual competency is important are more likely to believe that the department is understaffed and needs more personnel with different skills. This is consistent with the current situation. Education and title are common indicators for recruiting and evaluating talents and are linked to salary performance, so employees will try to improve their education level and title to get higher salaries. However, the actual ability required for some positions is not very related to the education or title. For example, the ability to adapt, communication, logical thinking, and comprehensive handling abilities that are required for administrative management positions are not significantly related to the education or title. Therefore, for those positions that require practical ability, although the hospital recruits many highly educated and high-titled talents, the leaders who value practical ability still see a shortage of staff. This finding also provides a direction for future recruitment and job hiring. For those positions that require practical ability or where department leaders focus more on practical skills, employees with better practical ability instead of having better educational backgrounds and titles should be deployed. These results indicate that the more individuals report that the hospital emphasizes competencies rather than titles, the more they tend to perceive insufficient numbers of staff and the need to hire different skill profiles of staff.

Concerning the impact on productivity, it is logical that respondents believe that the hospital with a higher level of productivity optimization has less staff shortage, (both on qualitative and quantitative dimensions), since productivity optimization level can basically reflect improvements in both staff quantity and quality. The findings pertaining to the correlation between productivity optimization and staffing quantitative and qualitative inadequacy are logical, since the reasoning about productivity optimization (in the way it was questioned) should mirror a sense of small margin to improve in both achieving the right number of employees and the right profile in recruitment. Study 1 examines productivity in four specific observed variables: the degree of human resource potential, absenteeism, motivation, and overtime. A productive organization is one in which people are working to their full potential, employees are highly motivated, absenteeism is low, and overtime is moderate. On the contrary, if employees are not highly motivated and often work overtime, section leaders in this state of production tend to feel that there are not enough staff.

Regarding the impact on decision-making, department heads who tend to believe that staffing decisions should be decentralized to their sections are more likely to believe that quantitative staffing is inadequate. Although the literature tends to favor decentralization in HRM as a measure of increasing the departmental leaders’ engagement with the overall HR decisions, findings concerning centralization versus decentralization in staffing showed that centralization seems to be helpful in avoiding understaffing or overstaffing. This is also logical because each department will struggle to increase existing resources and may not be aware of the needs of other departments. Only from a central position is it possible to understand where more resources for some become excessive, especially due to the lack of resources for other departments. An interesting finding is the lack of relation between this and qualitative staffing since some departments may be better at determining which qualitative profile should be hired and other departments are not as good at a centralized decision. This justifies why no significant association was found.

The findings also supported the second and third hypotheses, thus indicating that there is a negative relationship between quantitative staffing inadequacy and perceived team performance. This is consistent with the mainstream findings that understaffing leads to an increased workload of employees (75, 76), which in turn causes fatigue, negative emotions, and decreased productivity, ultimately leading to a decrease in team performance (77, 78).

This study used a 5-point Likert scale developed by Conger et al. (72) to test the team leader’s perception of performance, which better reflects the actual situation of team performance because the team leader is the leader closest to the production line and is most sensitive to the perception of team performance. Agyepong (37) found that overload changes employees’ psychological perceptions (i.e., internal psychological representations), which in turn decreases employee engagement and leads to a decrease in performance output. The model shows a negative relationship between quantitative staffing inadequacy and employee engagement and that quantitative staffing inadequacy exerts a negative indirect effect on team performance via work engagement. In line with most research, we can conclude that work engagement is a key issue in managing healthcare professionals (79).

It is clear that quantitative understaffing is not the only factor that affects team performance. Qualitative understaffing is also an active factor affecting team performance (80). Based on this theory, the research aimed to figure out the pathways through which qualitative understaffing could affect team performance. Findings did show that the indirect effect of quantitative staffing inadequacy on team performance via work engagement interacts with qualitative staffing inadequacy in such a way that when qualitative staffing inadequacy is higher, the negative indirect effect is stronger, and when qualitative staffing inadequacy is lower, the negative indirect effect is weaker. This has important implications both for theory and practice.

As regards theory, our findings add to existing knowledge by extending the focus on quantitative understaffing and adding the qualitative dimension. The interaction found means that no research on staffing management is completed without focusing on both dimensions, especially because qualitative seems to play a leveraging role in the whole process.

In practice, departments with more skilled staff ensure a certain level of productivity, and the higher productivity can mask to some extent the decline in performance due to inadequate quantitative staffing levels. These departments tend to achieve their work goals, have better performance output, and do not experience so strongly the need to recruit. Conversely, if departments lack skilled staff, more staff will be needed to accomplish the stated work objectives, in which case the qualitative understaffing further amplifies the decline in team performance due to the quantitative understaffing.

The model testing shows that staffing management policies indeed affect both qualitative and quantitative understaffing (department leaders’ common perception of department understaffing) and that staffing profiles (both qualitative and quantitative) in turn have impacts on team performance in a variety of ways. In other words, under the moderation of staffing management policies, human resource allocation (both qualitative and quantitative understaffing) has an impact on team performance.

A literature review conducted by Gile et al. explored the impact of hospital human resource management strategies on hospital effectiveness, which found that most studies focused on the effectiveness of employees and institutions, and there was little literature on the team and patient outcomes (81). The research gap motivated the conceptual model. The review of the literature revealed us that few studies have incorporated both the shortage of hospital staff and the lack of staff with the right skills into the theoretical model, i.e., the quantitative and qualitative staffing dimensions. This is an important research gap because focusing on only one of these dimensions is missing the point, as it is unreasonable to focus only on having the right ratio of employees without consideration for their competencies and other features that impact the quality and performance of their work, but it is also unreasonable to focus only on such competency profiles without considering the workforce ratios and staffing needs.

A study was conducted to test the conceptual model based on an overview of existing HRM policy studies. This model proposes five corresponding hypotheses. To summarize: Hypothesis 1: Staffing management policies impact the level of quantitative and qualitative staffing inadequacy; Hypothesis 2: Quantitative staffing inadequacy is negatively associated with perceived team performance; Hypothesis 3: Quantitative staffing inadequacy is negatively associated with work engagement; Hypothesis 4: Quantitative staffing inadequacy exerts a negative indirect effect on team performance via work engagement; and Hypothesis 5: Qualitative staffing inadequacy has a modulating effect upon the indirect effect stated as shown by interaction effects where inadequacy hampers the indirect effect while adequacy enables or increments it. Ultimately, the most central hypothesis is the last one that proposes a complex moderated mediation model.

As stated, the topic is complex, and such complexity stems also from the interaction of multiple people involved in policymaking, management, and employees themselves. Therefore, three versions of a questionnaire were developed and intended to collect data from three different important stakeholders: the first targeted employees’ perceptions of staff management policies and employee engagement. The second one targeted departmental leaders’ perceptions of qualitative and quantitative staffing inadequacy in the department. The third one was intended to survey team leaders’ perceptions of departmental performance outputs. The questionnaire survey was carried out at Shenzhen Maternity and Child Healthcare Hospital. The questionnaires were distributed through paper questionnaires and on-site collection, and the participants included staff at various levels in the hospital. After completing the questionnaire collection, valid questionnaires were obtained through certain screening procedures, and the collected data were used to test the model. Findings suggest that staffing management policies do affect qualitative and quantitative understaffing in three main ways: 1. The more individuals report that the hospital emphasizes competencies instead of titles, the more they tend to perceive insufficient numbers of staff and the need to hire staff with different skill profiles. 2. The more individuals perceive that the hospital has optimized its productivity, the less they think the hospital is lacking staff and lacking the right skills profile. 3. The more individuals perceive that the hospital is decentralizing staffing decisions to the departmental level, the more they think there is quantitative staff inadequacy. Moreover, the findings also show that quantitative understaffing is negatively related to team performance and employee engagement and that employee engagement plays a mediating role in the relationship between quantitative understaffing and team performance. The indirect effect of quantitative staffing inadequacy on team performance via work engagement interacts with qualitative staffing inadequacy in such a way that when qualitative staffing inadequacy is higher, the negative indirect effect is stronger, and when qualitative staffing inadequacy is lower, the negative indirect effect is weaker. To date, few studies have discussed the influences of staffing management policies on both employees’ perceptions and manager/departmental leaders’ perceptions. Therefore, this study may provide a better understanding of the current status of human resource management in Chinese hospitals and a basis for future comparative human resource management studies (74).

This study innovatively introduced qualitative and quantitative understaffing variables to test how staffing management policies can restrict staffing adequacy in a healthcare institution in Shenzhen, China, and how qualitative and quantitative staffing inadequacy affect team performance. The results reveal the important mechanism of the impact of staffing management policies and quantitative and qualitative personnel shortages on team performance, which has rich theoretical value.

In general, compared with the existing academic studies, this thesis innovatively establishes five key dimensions to describe the staffing management policy, helping scholars and managers to have an in-depth understanding of hospital human resource allocation. In addition, this study innovatively establishes a theoretical model for the impact of variables such as hospital staffing management policies and quantitative and qualitative staffing inadequacy on team performance, which enriches the understanding of existing research on the mechanisms affecting healthcare team performance and effectively supplements the literature related to healthcare HRM.

### Insights on personnel management

By testing Hypothesis 1: staffing management policies impact the level of quantitative and qualitative staffing inadequacy, we can conclude that leaders who tend to believe that practical skills are important tend to believe that the department is understaffed and needs more people with different skills. Medical institutions should pay more attention to the actual ability of employees in recruitment, selection, and promotion, especially in some positions that require actual ability. For example, the actual ability required for administrative positions may involve the ability of adaptation, communication, logical thinking, and comprehensive processing ability and break the stereotypical thinking of appointing talents based on academic qualifications and titles. When establishing a talent appraisal system, it is also important to fully consider the actual abilities of employees as an important aspect of appraisal.

We found that those department directors who tend to believe that staffing decisions should be delegated to their departments are more likely to believe that the quantity of staff is inadequate. As a public hospital, strategic decisions such as hospital development planning and human resource planning should be made centrally at the hospital level, and the current human resource scale in Chinese hospitals should also be controlled in total. For example, certain local documents stipulate that tertiary maternity and child healthcare institutions are configured according to a man-bed ratio of 1.7:1. At the departmental level, the production organization system should be continuously optimized to reduce overtime and improve staff motivation and satisfaction. We found in our study that respondents felt that hospitals with higher levels of productivity optimization were less short-staffed, which gives us an insight that when departments give feedback about the shortage of staff, they may be able to improve productivity and make up for the shortage of staff by improving the organizational production system, and the optimization should be based on an in-depth study of the department’s operation and human resource allocation. Through joint efforts at the hospital level and department level, a lean and efficient talent team can be established to provide a talent guarantee to promote the high-quality development for public hospitals.

### Research limitations

Like all studies, ours also has limitations that highlight potential areas for future research.

First, the research data of this study are all from a single medical institution in Shenzhen, China, and the data from one medical institution may not be universal. This cannot reflect the impact of the staffing management policies of all parts of China or other countries. In addition, we lack robust tests for the conclusions of the model, which could have an impact on the universality of the results of the experiment. However, we believe that these understaffing challenges are universally reported and that the requirements to overcome these challenges should also be common across hospitals.

Second, during the qualitative study, we identified five dimensions of staffing management policies (such as decision-making, data management, etc.); based on this categorization, a 13-item scale was produced, and its psychometric quality was ascertained. However, the selection of these dimensions may be subjective, which cannot fully summarize the staffing management policy, and may have a deviation in the research results. Still, the scale was subjected to validity and reliability testing, which encouraged its possible use in other settings.

Third, in the quantitative research of this experiment, utilizing work engagement as a mediator between quantitative staffing adequacy interaction and qualitative staffing adequacy in explaining team performance may overlook other important mediators, or there may be additional intervening variables that require further research.

### Research prospects

The model proposed by us demonstrates that staffing management policies indeed affect both qualitative and quantitative understaffing (department leaders’ common perception of department understaffing) and that staffing profiles (both qualitative and quantitative) in turn have impacts on team performance in a variety of ways. This is helpful to better design staffing management policies and increase the staffing fit so to improve team performance.

In the future, data can be collected from medical institutions all over the country and even the world (not limited to Shenzhen), and robust tests on the conclusions drawn from the model can be conducted to verify the rationality and universality of the experimental conclusions. This can also be extended to other international settings. In the construction of the conceptual model, one may be able to further find new intermediary variables (not just work engagement) or find the synergy of multiple intermediary variables and use these variables to link the relationship between quantitative and qualitative staffing adequacy, to further explain the impact on team performance.

In general, we hope that the measures developed in this research and the findings presented here will encourage further attention to these issues in the future as we seek to better understand and theorize staffing management.

## Data Availability

The raw data supporting the conclusions of this article will be made available by the authors, without undue reservation.
